# Computing the *Dynamic* Supramolecular Structural Proteome

**DOI:** 10.1371/journal.pcbi.1005290

**Published:** 2017-01-19

**Authors:** Ruth Nussinov, Jason A. Papin, Ilya Vakser

**Affiliations:** 1 Cancer and Inflammation Program, Leidos Biomedical Research, Inc., Frederick National Laboratory for Cancer Research, National Cancer Institute, Frederick, Maryland, United States of America; 2 Sackler Institute of Molecular Medicine, Department of Human Genetics and Molecular Medicine, Sackler School of Medicine, Tel Aviv University, Tel Aviv, Israel; 3 University of Virginia, Charlottesville, Virginia, United States of America; 4 Center for Computational Biology, The University of Kansas, Lawrence, Kansas, United States of America

Cells execute their functions through protein interactions. The pathways they link are neither discrete nor spatially separated, as typically depicted in cellular diagrams. Cellular diagrams are useful; however, they neglect the physical structure of cell signaling. In reality, functions are shaped by molecular transitions between small—and large—supramolecular assemblies. Even though they are preorganized, they consist of clusters that are loose and dynamic. Importantly too, they are often anchored in the membrane and interact with scaffolding proteins and the cytoskeleton. Their continuum may physically span the cell. Indeed, efficient, productive, and reliable cell signaling can only take place through transient and cooperative protein–protein interactions, not through stochastic, diffusion-controlled processes. Despite this, current computational approaches to the modeling of the structural proteome still do not fully account for the in vivo, real physical cell organization. The enigmas of the assembly sizes and dynamic conformational distributions—and the diverse cellular environments that influence them—present daunting challenges, which we have only begun to address.

How will we then compute the realistic structural proteome in the next decade? How will we overcome the challenges that we confront, and which methods should we develop to meet them? Clearly, our views of protein structure and function have undergone a revolution. We no longer believe that a protein exists in only two distinct (active and inactive) states. We now recognize that even though a specific function is executed by a distinct active state, proteins (and other bio-macromolecules) exist in ensembles of states. The structure–function paradigm that now dominates molecular biology was inspired by physics and chemistry, which stipulate that even living things must abide by the laws of quantum mechanics and structural chemistry. This paradigm argues that biomolecules should be viewed—and described—statistically, not statically. Though challenging, eventually, to realistically capture the functional versatility and model the working proteome, we must consider conformational ensembles and their allosteric shifts, which result in changes to the populations of the conformations. Moreover, within this framework, the heterogeneous cellular environments, as well as allosteric covalent post-translational modifications, cannot be overlooked. Have we indeed treated the structural proteome as such in our computations?

Determining the structures of protein assemblies has long been a vastly important aim of structural biology. The problem is challenging: a pair of protein structures can interact by complementary patches of surfaces. The patch size and identity are unknown, and it is difficult to assess which patches on one protein interact with which patches on the other. In principle, each patch of surface of one protein needs to be matched with each of the other, while at the same time making sure that no other part of each of the proteins penetrates the volume occupied by the other. The problem is compounded by protein flexibility. Proteins are not static sculptures; instead, they are dynamical objects that are always interconverting between a variety of structures with varying energies. Association of two proteins (or biomolecules) involves matching of chemical and geometrical signatures out of the enormous number of possible ones for all conformers.

The scientific community took up this challenge. Over 45 years, scientists have developed algorithms to predict, refine, and score molecular interactions, starting with Harold Scheraga’s landmark paper in 1972 [1] (remarkably, on ligand-receptor docking) and followed by the first two protein–protein docking papers by Shoshana Wodak and Joel Janin [2] and Jonathan Greer and Bruce Bush [3] in 1978. Subsequent highly influential works early on were real-time graphics developed by Bob Langridge and Michael Connolly [4] and DOCK, a geometrical approach based on combinatorial distance geometry by Tack Kuntz [5]. The revolutionary Fast Fourier Transform (FFT) method, published in 1992 by Ephraim Katchalski-Katzir and coworkers [6], was similarly purely geometrical but much faster, thus allowing computationally feasible exhaustive search of the full six-dimensional rigid-body docking space. Since its inception, this algorithm has formed the foundation for numerous docking strategies. To date, many FFT-based variants and pieces of software have been written and applied. In parallel, in 1991, Ruth Nussinov and Haim Wolfson [7] published the geometric hashing (GH) method, an approach originally developed for object recognition problems in computer vision. Unlike the FFT, the GH does not carry out an exhaustive six-dimensional search; instead, it introduces an indexing approach based on transformation invariant representations toward efficient recognition of partial structures. Since these pioneering works, a number of additional successful strategies were published, including HADDOCK (High Ambiguity Driven biomolecular DOCKing) [8], based on biochemical and/or biophysical information; PRISM (Protein Interactions by Structural Matching) [9], which is based on template interface motifs; and more. Refinement and scoring have also improved substantially, resulting in a fairly robust ranking of the predicted solutions [10–13]. Critically, major experimental initiatives provided a growing number of high resolution (as well as lower resolution) structural data, which have been exploited by these algorithmic strategies [14].

Despite these advances, at the dawn of 2017, the challenge is still there. Which directions to pursue in the next decade? Development of algorithms to increasingly, efficiently, and reliably exploit the emergence of data from electron microscopy (EM) maps to obtain small and large complexes; accounting for ensembles of states, which can also be helped by EM data as well as efficient sampling methodologies; developing of next-generation, multiscale simulation protocols for large assemblies with the ability to account for the different cellular environment; and more are all possible avenues of exploration. At the same time, there needs to be a sufficient level of detail to permit us to account for the effects of mutations. So far, structural modeling of protein complexes has largely assumed that the proteins interact in dilute solution. However, adequate representation of the crowded in vivo environment has to deal with much more complex systems, requiring coarse-graining and simplifications. Closing the gap between coarse-grain and atomic resolution methodologies will require development of multiscale approaches to introduce higher resolution structural information into large-scale modeling protocols. Put together, such approaches would potentially provide an integrated, self-consistent model of the interactome in vivo.

Modeling the proteome forms the basis for diverse computational and experimental endeavors. Among these are prediction of function and elucidating functional mechanisms; figuring out signaling pathways in the cell and communication routes; drug discovery; and elucidation of disease, catalysis, viral packaging, and more. *PLOS Computational Biology*, the premier computational biology journal, aims to help spearhead and map initiatives toward the development of transformative tools and methodologies (and their applications) to foster scientific advancements in the next decade. It aims to empower biological computations as a leading force in the life sciences. *PLOS Computational Biology* can drive research to fill fundamental knowledge gaps; *PLOS Computational Biology* aspires to serve as a forum that impels, formulates, defines and articulates, and, finally—conjointly with the experimental sciences—advances and guides to achieve these aims.
